# Mutations in GDF5 Reveal a Key Residue Mediating BMP Inhibition by NOGGIN

**DOI:** 10.1371/journal.pgen.1000747

**Published:** 2009-11-26

**Authors:** Petra Seemann, Anja Brehm, Jana König, Carsten Reissner, Sigmar Stricker, Pia Kuss, Julia Haupt, Stephanie Renninger, Joachim Nickel, Walter Sebald, Jay C. Groppe, Frank Plöger, Jens Pohl, Mareen Schmidt-von Kegler, Maria Walther, Ingmar Gassner, Cristina Rusu, Andreas R. Janecke, Katarina Dathe, Stefan Mundlos

**Affiliations:** 1 Research Group Development and Disease, Max-Planck-Institut für Molekulare Genetik, Berlin, Germany; 2 Berlin-Brandenburg Center for Regenerative Therapies, Charité – Universitätsmedizin Berlin, Berlin, Germany; 3 Institut für Medizinische Genetik, Charité – Universitätsmedizin Berlin, Berlin, Germany; 4 Freie Universität Berlin, Berlin, Germany; 5 Institute of Anatomy, Department of Anatomy and Molecular Neurobiology, Universitätsklinikum Münster, Münster, Germany; 6 Lehrstuhl für Physiologische Chemie II, Theodor-Boveri-Institut für Biowissenschaften (Biozentrum) der Universität Würzburg, Würzburg, Germany; 7 Department of Biomedical Sciences, Baylor College of Dentistry, Texas A&M University System Health Science Center, Dallas, Texas, United States of America; 8 Biopharm GmbH, Heidelberg, Germany; 9 Department für Kinder- und Jugendheilkunde, Medizinische Universität Innsbruck, Innsbruck, Austria; 10 Medical Genetics Department, University of Medicine and Pharmacy, Iaşi, Romania; 11 Sektion für Klinische Genetik, Medizinische Universität Innsbruck, Innsbruck, Austria; Stanford University School of Medicine, United States of America

## Abstract

Signaling output of bone morphogenetic proteins (BMPs) is determined by two sets of opposing interactions, one with heterotetrameric complexes of cell surface receptors, the other with secreted antagonists that act as ligand traps. We identified two mutations (N445K,T) in patients with multiple synostosis syndrome (SYM1) in the BMP–related ligand *GDF5*. Functional studies of both mutants in chicken micromass culture demonstrated a gain of function caused by a resistance to the BMP–inhibitor NOGGIN and an altered signaling effect. Residue N445, situated within overlapping receptor and antagonist interfaces, is highly conserved among the BMP family with the exception of BMP9 and BMP10, in which it is substituted with lysine. Like the mutant GDF5, both BMPs are insensitive to NOGGIN and show a high chondrogenic activity. Ectopic expression of BMP9 or the GDF5 mutants resulted in massive induction of cartilage in an in vivo chick model presumably by bypassing the feedback inhibition imposed by endogenous NOGGIN. Swapping residues at the mutation site alone was not sufficient to render Bmp9 NOG-sensitive; however, successive introduction of two additional substitutions imparted high to total sensitivity on customized variants of Bmp9. In conclusion, we show a new mechanism for abnormal joint development that interferes with a naturally occurring regulatory mechanism of BMP signaling.

## Author Summary

The development of the human skeleton is regulated by intricate signaling pathways involving secreted molecules that bind to cell surface receptors to elicit a response in the target cell. Bone morphogenetic proteins (BMPs) are an important part of this process. Their signaling capacity is regulated on several levels including the extracellular space where inhibitors such as Noggin prevent BMPs from binding to their cognate receptors. We here describe that specific mutations of a single amino acid in GDF5, a member of the BMP family, cause congenital fusion of joints. Investigating the effect of the mutation in detail, we can show that the mutant GDF5 is no longer inhibited by Noggin, thus providing a functional explanation for the patients' phenotype. Furthermore, we show that the mutated residue (N445) is conserved throughout the BMP family, with the exception of BMP9 and BMP10, that carry the same amino acid at this position as the mutant GDF5. Both are, just as the mutant, resistant to inhibition by Noggin. Variants of BMPs that are insensitive to antagonists may induce bone formation more effectively, providing a source for effective, low-dose therapeutics for clinical applications.

## Introduction

Bone Morphogenetic Proteins (BMPs) and the related Growth & Differentiation Factors (GDFs) are phylogenetically conserved signaling proteins that belong to the Transforming Growth Factor beta (TGFβ) superfamily. Originally identified for their ability to induce bone, they were subsequently shown to be involved in multiple aspects of body patterning and morphogenesis [1]–[2]. Mutations in BMPs and their receptors can cause a wide variety of congenital and postnatal diseases [3]–[4]. Despite their different functions, all BMPs share a common signaling mechanism. They are translated as precursor proteins consisting of a prodomain, which is released proteolytically by members of the subtilisin-like proprotein convertase family [5], which is important to activate signaling that is conferred through the mature domain [6]. The highly conserved mature domain is characterized by seven cysteine residues. Six of them form an intramolecular cysteine knot whereas the fourth of the seven cysteines is important for the dimerization of the BMP monomers [7]. BMPs are secreted peptides that act as homo- or heterodimers and bind to two different types of membrane-spanning serine/threonine kinase receptors, the type I BMP receptors (ACVRL1, ACVR1, BMPR1A, BMPR1B) and type II BMP receptors (BMPR2, ACVR2). Binding of BMPs to heterotetrameric receptor complexes activate the SMAD as well as other intracellular signaling pathways, like the MAPK pathway [8]. BMP signaling is precisely regulated by a large number of antagonists, which act extracellularly, on the membrane level as well as intracellularly [9]. Noggin (NOG; MIM *602991), one of the best characterized extracellular BMP antagonists, was first isolated as a dorsalizing factor secreted by Spemann's organizer of Xenopus embryos [10] and later shown to be required for patterning of the neural tube and in skeletogenesis [11]–[12]. The crystal structure of NOG bound to BMP7 provided the structural basis of inhibition of BMP signals, revealing the clamp-like grip of the covalently linked homodimer on the homodimeric ligand that blocks both pairs of receptor binding interfaces [13]. The importance of NOG for the development of joints was shown by the identification of mutations in *NOG* in patients with symphalangism (SYM1, MIM #185800) and multiple synostosis syndrome (SYNS1, MIM #186500) [14]. We and others have shown that certain mutations in *GDF5* (also known as CDMP1; MIM *601146) are also associated with SYM1 and SYNS1 [15]–[18]. SYM1/SYNS1 associated mutations result in over-active GDF5 due to altered receptor binding specificity. In contrast, loss of function mutations in *GDF5* result in brachydactyly types C or A2 (BDC, MIM #113100; BDA2 MIM #112600) [6], [19]–[20]. Homozygous loss of function mutations cause acromesomelic chondrodysplasia of the Grebe (MIM #200700), Hunter Thompson (MIM #201250) or Du Pan (MIM #228900) types, conditions characterized by extremely short limbs and digits [21]. A recent and comprehensive review on the respective genotype-phenotype correlations has just been compiled and published [4]. Here, we describe two novel *GDF5* point mutations (N445K and N445T) that are associated with a pronounced form of SYNS1. Functional characterization of the mutants demonstrated increased biological activity when compared to wtGdf5 due to a resistance against inhibition by Nog. The importance of the N445 residue for NOG function was further substantiated by identification of two other BMPs, BMP9 and BMP10, that share the same replacement at this site and that are also insensitive to inhibition by NOG.

## Results

### Mutations at position N445 in GDF5 cause multiple synostosis syndrome

**Figure 1:**
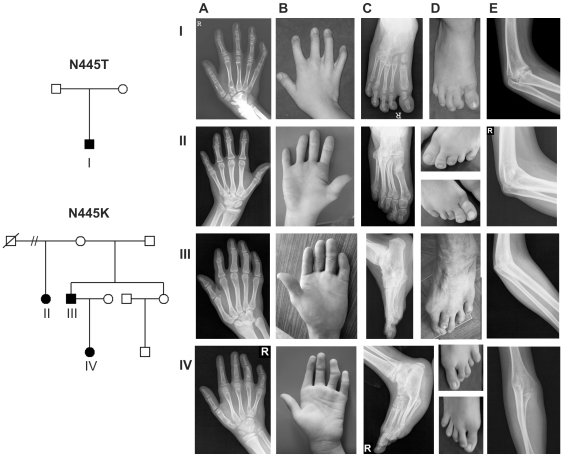
GDF5-missense mutations at position N445 are associated with multiple synostosis syndrome. Pedigrees are shown on left. Clinical phenotypes associated with *GDF5* mutations N445T (I) or N445K (II, III, IV) are shown in (A–E). Pictures in each horizontal line belong to one patient. (I) N445T mutation in a 5 year old boy; (II), (III) N445K mutation in affected sibs, both adults; (IV) 9 year old girl with the N445K mutation. (A) Hand radiographs show fusion and abnormal configuration of carpal bones as well as proximal symphalangism of fingers II to V in all affected. Short first metacarpal bones are present in patient I and II, shortened first proximal phalanges are visible in patient II. (B) Clinical pictures display the brachydactyly of fingers II to V and missing flexion creases (see II, III). Some distal phalanges are hypoplastic to variable degree (I). (C, D) Feet are similarly affected with fusion of tarsal bones, proximal symphalangism, shortened toes or hypoplastic toenails. (E) Synostosis of humerus and radius leading to a stiffened elbow joint.

We identified a heterozygous missense mutation c.1335T>G leading to an exchange of the hydrophilic asparagine to basic lysine (p.N445K) in three family members with SYNS1. The mutation segregated in an autosomal dominant manner. The clinically unaffected mother of two affected children did not show the p.N445K mutation arguing for the presence of a germline mosaicism. In addition, we identified a de novo asparagine to threonine (p.N445T) mutation (c.1334A>C) in another patient with SYNS1. All four affected patients exhibited the characteristic features of severe SYNS1 (Figure 1). The radiographs showed bilateral fusions of carpal and tarsal bones as well as proximal symphalangism in fingers and toes. Distal phalanges of fingers and toes II to V were hypoplastic and some nails appeared small. Fusions between humerus and radius were present in all four patients leading to fixation of the elbow joints in a flexed position. Mild cutaneous syndactyly was present in some individuals. The phenotype was congenital and appeared to be non-progressive.

### N445 co-localizes within the BMP type I receptor and NOG interaction sites

**Figure 2:**
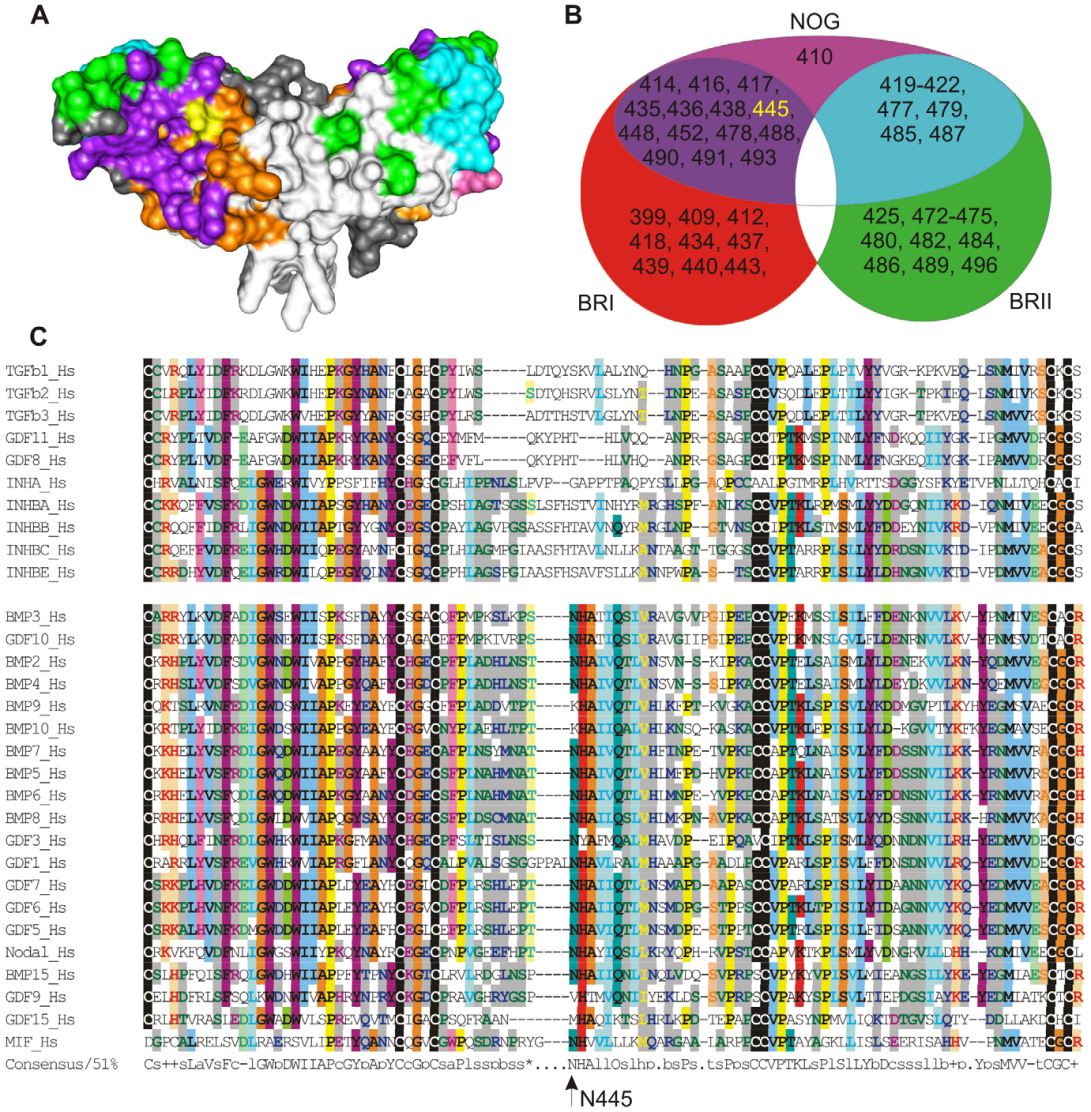
Location of N445K,T mutations within the GDF5 dimer. (A) GDF5 dimer (light, dark grey) linked via a disulfide bridge (PDB: 3EVS). Amino acid residues predicted to form the BMP type I receptor interface are indicated in orange, the BMP type II receptor interface in green, and the NOG interface in magenta. Amino acids that bind to BMPRI and NOG are indicated in violet, amino acids important for BMPRII and NOG binding are indicated in cyan. Amino acid N445 is indicated in yellow. Note, the position N445 is within the receptor interaction site as well as within the NOG interaction site. (B) Venn diagram indicates predicted GDF5 amino acids important for NOG and receptor interaction. (C) The mutated asparagine of the GDF5 N445K,T mutants is located in the region between the third and fourth conserved cysteine of the mature domain of the TGFβ superfamily, also called the “wrist” epitope of the hand-like BMP/GDF monomer. Note that the divergent BMP9, BMP10 pair share a lysine at this position.

A 3D structural model of GDF5 in complex with BMPR1B (PDB 3EVS; [22]) with homology-based binding epitopes for the type II receptors superimposed on that for NOG shows that asparagine 445 co-localizes within both the type I receptor and the NOG interaction sites (Figure 2A and 2B). Sequence alignments showed that the N445 residue is conserved in the related BMP/GDF group, with the exception of BMP9 (K372), BMP10 (K368), GDF9 (V398) and GDF15 (M253) (Figure 2C).

### Gdf5 mutants N445K and N445T render Gdf5 insensitive to Nog

**Figure 3:**
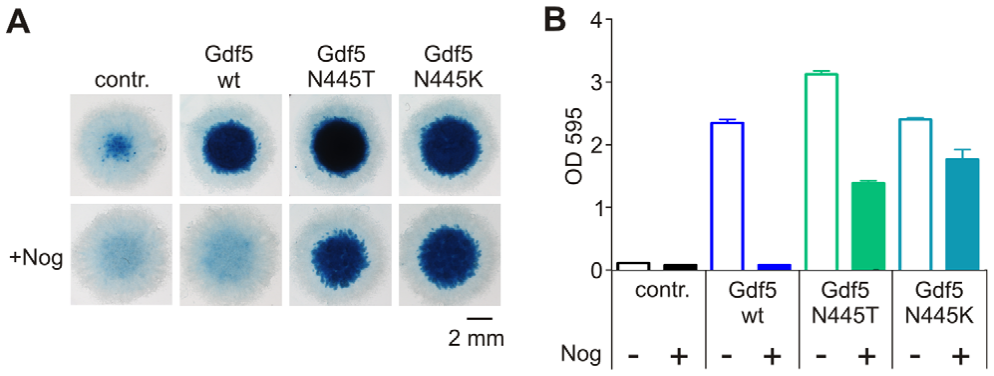
Gdf5 mutations N445T and N445K are largely Nog-insensitive in chicken micromass cultures. Chicken limb bud micromass cultures were retrovirally infected to express the indicated proteins and stained with Alcian blue after five days of cultivation. (A) WtGdf5 and Gdf5 mutants N445T and N445K strongly stimulate chondrogenesis. Co-infection with Nog inhibits this effect in the control and wtGdf5. In contrast, the mutants Gdf5 N445T and N445K are only slightly inhibited by Nog. (B) Quantification of Alcian blue staining after extraction and photometric measurement at OD_595_ (n = 4).

Retroviral expression of Gdf5 in micromass cultures induces whereas Nog inhibits chondrogenesis, which can be quantified by Alcian blue staining of the extracellular matrix. The N445K and N445T mutants induced chondrogenesis to a similar extent as wtGdf5. Co-expression of Nog prevented induction of chondrogenesis by wtGdf5, but had only a minor effect on the Gdf5 mutants, demonstrating the critical role of the asparagine residue in the interaction of Gdf5 with Nog (Figure 3A and 3B).

### BMP9 and BMP10 are naturally insensitive to inhibition by NOG

**Figure 4:**
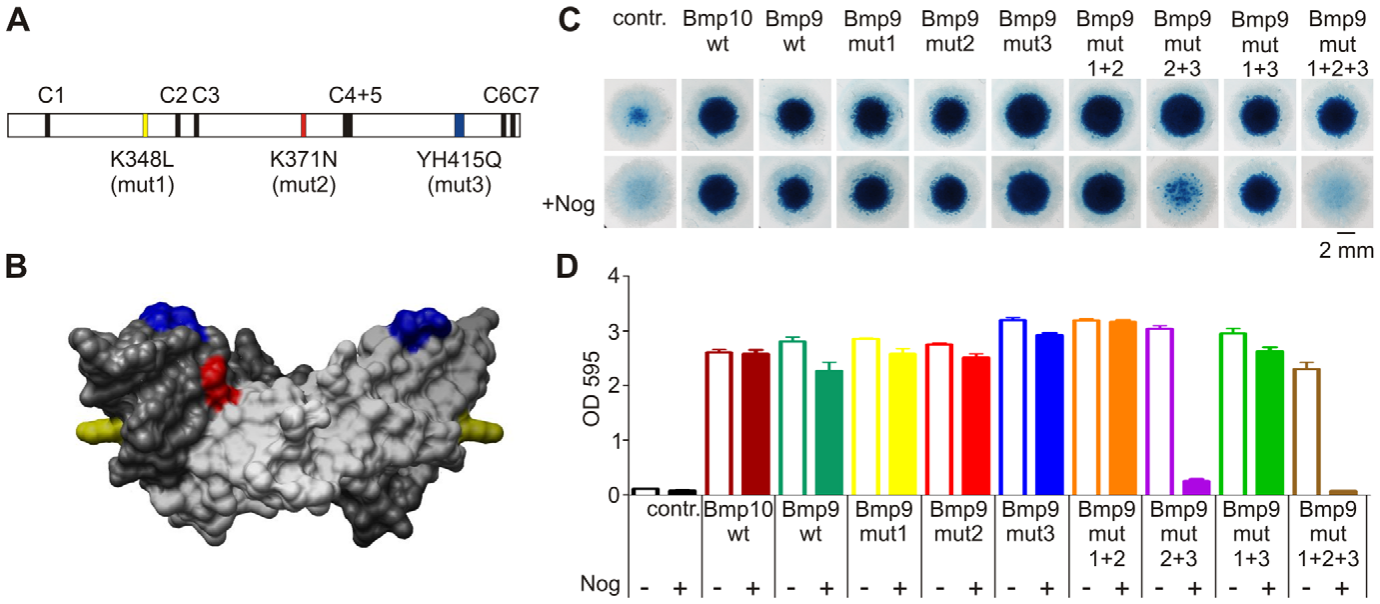
Bmp9 and Bmp10 are not inhibited by Nog. Chicken limb bud micromass cultures were retrovirally infected to express the indicated proteins and stained with Alcian blue after five days of cultivation. (A) Schematic overview of the mature domain of mouse Bmp9. Conserved cysteines marked in black. (B) 3D surface model of a BMP9 dimer (light grey and dark grey; PDB∶1ZKZ) linked via a disulfide bridge. Mut2 (K371N) represents the homologous mutation site to GDF5 N445T and is indicated in red. Mut1 (K348L, yellow) and mut3 (YH415Q, blue) are additional sites, which were predicted to be important for the interaction of GDF5 with NOG. (C) Expression of wtBmp9 and wtBmp10 in chicken micromass strongly induce chondrogenesis. This effect cannot be inhibited by co-expression of Nog. The Bmp9 mutants mut1 (K348L), mut2 (K371N), mut3 (YH415Q), mut1+2, and mut2+3 are similar to wtBmp9. In contrast, the combination of K371N and YH415Q (mut2+3) results in partial inhibition by Nog. The combination of all three mutations (mut1+2+3) leads to a complete inhibition by Nog. (D) Quantification of Alcian blue staining after extraction and photometric measurement at OD_595_ (n = 4).

BMP9 and BMP10 share the same amino acid residue at this position as one of the SYNS1- associated mutants. We analyzed Bmp9 and Bmp10 in the micromass system and observed an even stronger chondrogenic effect than for wtGdf5, indicating that they are potent inducers of chondrogenic differentiation. Co-expression of Nog did not suppress chondrogenesis significantly, demonstrating that both ligands are naturally Nog-insensitive unlike most other Bmps. To test whether the substitution shared by Bmp9 and Bmp10 conferred resistance to Nog, we swapped the wildtype lysine residue of Bmp9 with asparagine (K371N), the residue shared by most Bmp family ligands, including Gdf5. This exchange was not sufficient to transform Bmp9 into a Nog-sensitive Bmp (Figure 4A, 4B, 4C, and 4D), indicating that additional residues must contribute to diminished binding. Hence two other dissimilar sites within the NOG interface, shown in schematic and 3D surface models of BMP9 (Figure 4A and 4B), were targeted for exchange. Introducing a second swap (YH415Q) at a contact with the NOG N-terminal extension or “clip” diminished biological activity substantially upon co-expression of Nog. A third successive mutation (K348L), located on the periphery of the type II receptor interface, rendered Bmp9 completely Nog-sensitive relative to the control, without significantly affecting the prochondrogenic potential of the custom variant in the absence of Nog (Figure 4C and 4D).

**Figure 5:**
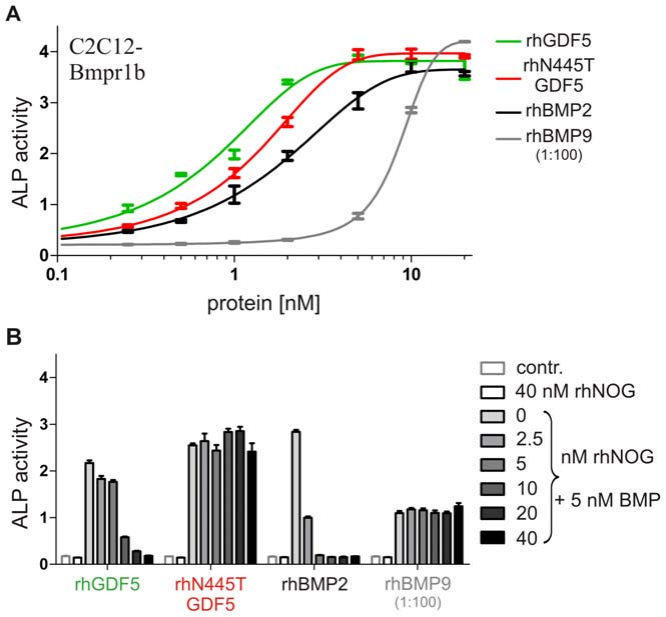
NOG insensitivity of rhN445T GDF5 in vitro. (A) ALP activity induced in C2C12 myoblastic mouse cells overexpressing the Bmpr1b receptor (C2C12-Bmpr1b) after three days of stimulation. All BMPs show dose-dependent induction of ALP activity. (B) Inhibition of rhBMP-induced ALP activity in the C2C12-Bmpr1b cell line by rhNOG. ALP activity was measured after co-stimulation of 5 nM rhGDF5, rhN445T GDF5, rhBMP2, and 0.05 nM rhBMP9 with up to 40 nM rhNOG (rhBMP9 up to 0.4 nM). RhBMP2 is strongly inhibited by rhNOG. In contrast to rhGDF5 the mutant rhN445T GDF5 shows no response to rhNOG inhibition.

To analyze the relative resistance to NOG more quantitatively, recombinant protein of one of the mutants (N445T) was produced and dose-dependent inhibition assays performed. For this C2C12 cells that stably express Bmpr1b, the high affinity type I receptor for GDF5, have been chosen because all analyzed proteins induced a significant amount of ALP activity, making it a convenient and reliable assay to analyze the inhibitory effect of NOG [16]. Dose response curves were generated to determine optimal concentrations for subsequent inhibition assays (Figure 5A). C2C12-Bmpr1b cells were incubated with rhGDF5, rhN445T GDF5, rhBMP2 or rhBMP9 in combination with rhNOG at increasing concentration and ALP activity determined after three days. Although rhBMP2 was completely inhibited by equimolar rhNOG, the apparent affinity of rhGDF5 for the antagonist was markedly less pronounced, requiring super-stoichiometric ratios to achieve total inhibition. Consistent with the effects in micromass, rhN445T GDF5 and rhBMP9 were unresponsive to rhNOG at any concentration (Figure 5B).

### Altered signaling effect of rhN445T GDF5

**Figure 6:**
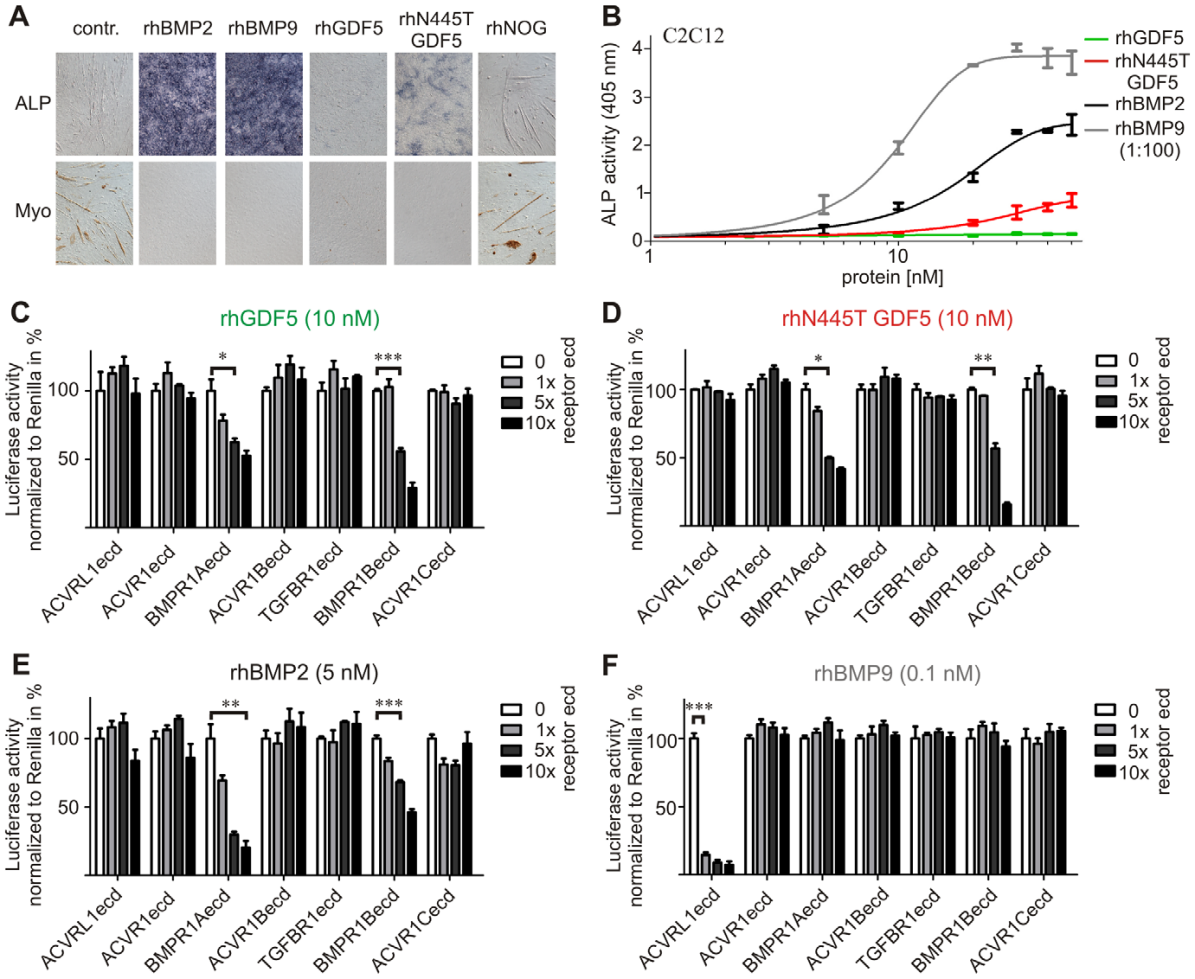
rhN445T GDF5 has a different signaling effect than rhGDF5. (A) Differentiation of myoblastic mouse cell line C2C12 was documented with alkaline phosphatase (ALP) staining (for osteoblasts) and immuno-myosin staining (for myoblasts) after five days of stimulation with 10 nM of the indicated recombinant proteins. RhBMP2 and rhBMP9 strongly induced ALP activity and inhibited myoblast differentiation, whereas rhGDF5 affected osteoblast differentiation only slightly while inhibiting myoblast differentiation. Interestingly rhN445T induced a moderate ALP activity and showed no detectable myosin staining. (B) ALP activity induced by stimulating the C2C12 with BMPs for three days. RhN445T, rhBMP2, and rhBMP9 showed dose-dependent induction of ALP activity whereas rhGDF5 had almost no ability to induce ALP activity. (E–G) Luciferase reporter assay of rhGDF5, rhN445T GDF5, rhBMP2, and rhBMP9 activity co-stimulated with soluble receptor extracellular domains (ecd) of the seven TGFβ superfamily type I receptors in C2C12 cells. rhGDF5, rhN445T GDF5 as well as rhBMP2 activity was competitively inhibited by BMPR1Aecd and BMPR1Becd, whereas luciferase activity induced by rhBMP9 was inhibited by ACVRL1ecd. Luciferase activity was normalized to Renilla. The lowest unstimulated value was determined as 0% and BMP stimulation without receptor ecd as 100%. Statistically relevant interactions were calculated with a two-tailed t-test and marked as: * p≤0.05, ** p≤0.01, *** p≤0.001.

RhGDF5 only slightly induced ALP activity, but effectively inhibited myogenic differentiation of the myoblastic cell line C2C12. In contrast, the rhN445T GDF5 mutant induced ALP activity in a dose dependent manner in C2C12 cells, which indicates an altered signaling effect (Figure 6A and 6B). To test possible alterations of the interaction between the N445T mutant and the type I receptor, we co-stimulated C2C12 cells with recombinant proteins with soluble ectodomains (ecd) of TGFβ superfamily type I receptors and used the luciferase reportergene assay as a read out (Figure 6C, 6D, 6E, and 6F). A direct comparison of rhGDF5 and rhN445T GDF5 revealed that addition of BMPR1Aecd as well as BMPR1Becd had a slightly stronger inhibitory effect on rhN445T GDF5 than on rhGDF5. As anticipated, rhBMP2 was almost completely inhibited by the ectodomain of rhBMPR1A and partially by the ectodomain of rhBMPR1B [23], whereas rhBMP9 was antagonized by the ectodomain of rhACVRL1 [24]. To identify possible differences between rhGDF5 and rhN445T GDF5 with respect to their binding affinities to the two BMP type I receptors BMPR1A and BMPR1B we analyzed them by using Biacore. However, no significant changes between rhGDF5 and rhN445T GDF5 could be observed in these experiments (Table 1).

**Table 1:** Biacore interaction analysis (n = 6) showed no binding differences for rhGDF5 and rhN445T GDF5 with BMPR1Aecd and BMPR1Becd.

	Apparent K_D_ [nM] for immobilized receptor ectodomains	
	BMPR1Aecd	BMPR1Becd
**rhGDF5**	17,0±4,6	1,1±0,2
**rhN445T**	19,5±5,5	1,2±0,3

### Increased biological activity of NOG-insensitive BMPs from bypassed feedback inhibition of Nog upregulation

**Figure 7:**
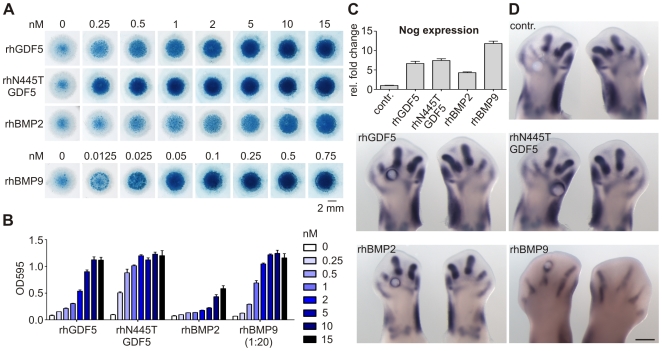
GDF5 mutant N445T has increased prochondrogenic activity. Stimulation of chicken micromass cultures with rhGDF5, rhN445T GDF5, rhBMP2, and rhBMP9 at day one for 72 h with the indicated concentrations. (A) Alcian blue staining of micromass cultures at day four. RhN445T showed a strong induction of chondrogenesis already at low concentrations. In comparison, rhBMP2 showed a strongly reduced and rhBMP9 the highest prochondrogenic activity. (B) Quantification of the Alcian blue staining, n = 3. (C) Semi-qRT-PCR for Nog after 12 h stimulation of one day old chicken micromass cultures with 5 nM rhGDF5, rhN445T GDF5, rhBMP2, and 0.5 nM rhBMP9. Stimulation with recombinant proteins induces a strong upregulation of endogenous Nog expression. (D) Whole mount in situ hybridizations showing *Nog* expression in chicken hind limbs after implantation of heparin beads soaked with 4 mM HCl + 0.2% BSA, 0.5 mg/ml rhGDF5, 0.5 mg/ml rhN445T GDF5, 0.5 mg/ml rhBMP2, and 0.25 mg/ml rhBMP9. Beads were implanted between HH stages 25–27 and HH29/30 (BMP9) and embryos were collected 20 h later. In contrast to the control limb, all beads soaked with BMP induced *Nog* expression in the periphery.

**Figure 8:**
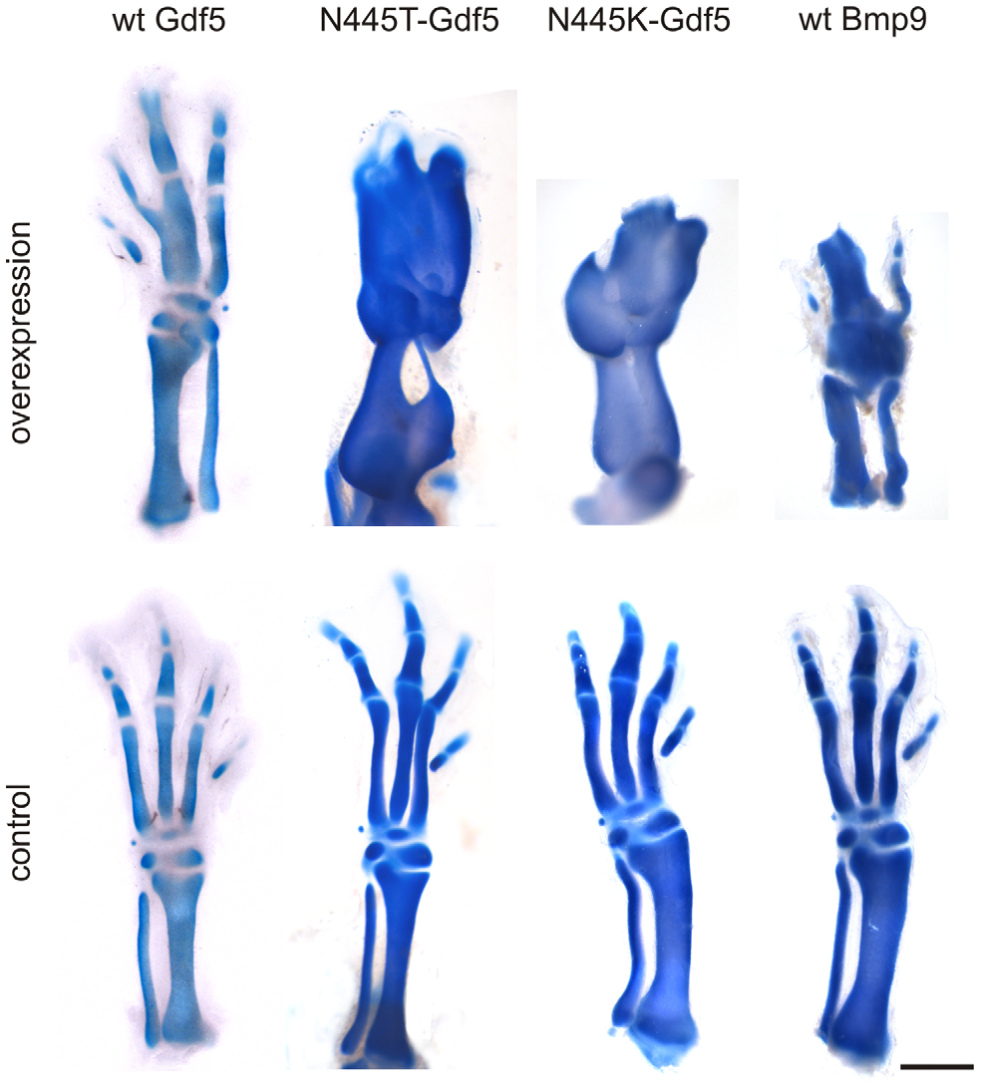
Gdf5 mutants N445T and N445K as well as Bmp9 cause strong chondrogenic differentiation when overexpressed in chicken limbs. Retroviral overexpression of indicated proteins after injection into chicken hind limbs at stage HH10. The contralateral hind limb was used as control. Embryos were harvested at day 7.5 and skeletal preparation stained with Alcian blue and Alizarin red. WtGdf5 lead to fusion of joints in the phalanges and metatarsals. In contrast, N445T and N455K Gdf5 as well as Bmp9 overexpression in vivo caused a severe phenotype with massive cartilage production leading to complete fusion of all skeletal elements of the limb and absence of interdigital mesenchyme. Scale: 1 mm.

Stimulation of micromass cultures with recombinant proteins revealed stronger chondrogenic differentiation with rhN445T GDF5 than with rhGDF5 or rhBMP2. Because of the high potency of rhBMP9, a 1∶20 dilution was employed to prevent saturation effects (Figure 7A and 7B). Quantitative real time PCR showed that treatment of micromass cultures with rhGDF5, rhN445T GDF5, rhBMP2 or rhBMP9 resulted in an upregulation of Nog (Figure 7C). Upregulation of Nog by exogenous rhBMP was also examined in vivo. Heparin beads soaked with recombinant protein were implanted into chicken limb buds and in situ hybridization for Nog was performed. Implantation of rhBMP9 soaked beads (0.5 mg/ml) at day four (HH25–27) resulted in early lethality (n = 40). To avoid this effect, the beads were implanted at a later stage (HH29/30) containing less protein (0.25 mg/ml). In agreement with the in vitro results, we observed a strong upregulation of endogenous Nog surrounding beads indicating that wildtype as well as mutant BMPs induce the expression of their inhibitor (Figure 7D). To further evaluate and compare activity of growth factors in vivo, we overexpressed wtGdf5, N445T Gdf5, N445K Gdf5 and Bmp9 in developing chicken hind limb buds. Overexpression of wtGdf5 caused a general thickening of skeletal elements, with fusions of joints as well as digits. Overexpression of N445K and N445T Gdf5 resulted in a much more severe phenotype. The entire limb morphed into a single cartilage element, without joints or even soft tissue. Injection of Bmp9 had a lethal effect; however the Bmp9-infected embryos that survived (7 out of 40) showed a phenotype similar to N445 Gdf5 mutants (Figure 8). Thus, the increased biological activity of the GDF5 mutants as well as BMP9 may arise in large part from insensitivity to endogenously upregulated Nog.

## Discussion

Transduction of temporally and spatially specific signals through the BMP pathway in a diverse array of cellular contexts is achieved in part by differential affinities of the receptors of the heterotetrameric-signaling complex for the array of ligands in the family. However, differential affinity to secreted inhibitors that act as ligand traps plays a reciprocal, equally important role in context-specific tuning of BMP signals. Here, we show that single point mutations (N445K,T) in the wrist epitope of the BMP-related ligand GDF5 lead to a resistance to Nog, and a subsequent dramatic increase in the intensity of the signaling output. Moreover, we show that wildtype BMP9 and BMP10, two divergent members of the family that harbor the asparagine for lysine substitution, are markedly resistant to Nog in limb bud micromass and C2C12 myoblast cell assays.

The GDF5 mutations N445K,T are associated with an inherited skeletal disease characterized by joint fusions in fingers, toes and elbows. This phenotype is similar to that of SYNS1, previously associated with hypomorphic alleles of *NOG*. SYM1, a less severe condition in which symphalangism is limited to the small interphalangeal joints of the fourth and fifth finger, is also caused by mutations in *NOG*. We have previously shown that a SYM1-like phenotype can also result from gain of function mutations in *GDF5* [15]–[16].

GDF5 mutations described here affect the wrist epitope, which is the polypeptide segment between the third and fourth conserved cysteine residue that is most divergent as shown in the multiple sequence alignment of TGFβ superfamily ligands (Figure 3). Because the wrist epitope is a key element of the type I receptor interface, the divergence may reflect the differential affinity of the superfamily of signaling ligands for their specific receptors. In addition, the diversity may reflect differential affinity for the array of secreted antagonists. For example, TGFβ ligands are deposited extracellularly as an inactive latent complex not regulated by secreted antagonists, whereas BMP/GDF ligands can bind with picomolar affinity, or evade inhibition almost entirely, as we have shown here for the interaction of NOG with the divergent BMP9, BMP10 pair [25]–[26].

Functional studies of the N445K,T mutations suggest a dual effect of the mutations - an altered signaling effect on the one hand and resistance to NOG on the other. Whereas the insensitivity of N445K,T mutations was clearly demonstrated in retroviral overexpression studies or by stimulation with recombinant proteins, the altered signaling effect could not be finally clarified on a molecular level. The fact that - in contrast to rhGDF5 - the rhN445T GDF5 mutant induced significant amounts of ALP in C2C12 cells prompted us to speculate on altered receptor specificity. This hypothesis was further analyzed by neutralization assays of different TGFbeta type 1 receptors and Biacore analyses. RhN445T GDF5 seemed to be slightly more sensitive to the inhibition by BMPR1Aecd and BMPR1Becd, but no significant changes of receptor affinities were detected. The different signaling effect might be explained either by different association and dissociation rates of the ligand-receptor complex or by the involvement of other co-receptors.

Interestingly, the reported R438L mutation in GDF5 lies within the same domain, i.e. the wrist epitope (type I receptor interface as well as NOG binding interface). Yet it results in altered receptor specificity with increased affinity to the BMPR1A, and not affecting the inhibition by NOG. The R438 position is not conserved within the BMP/GDF subgroup. We demonstrated that the rhR438L GDF5 mutant has a higher affinity for the BMPR1A receptor than rhGDF5, giving this mutant BMP2-like properties. BMP2 is also inhibited by NOG, which indicates that this amino acid change seems to have an effect on the receptor specificity only, but otherwise it is well tolerated by the BMP antagonist NOG.

The similarity of the resulting phenotypes caused by either loss of function NOG mutations or activating mutations in GDF5 argues in favour of GDF5 representing the most important target for NOG during the critical phase of joint development and indicates that SYM1/SYNS1 are associated with an increased biological activity of GDF5.

The crystal structure of the NOG-BMP7 complex showed that NOG, a covalently linked homodimer, inhibits BMP signaling by blocking the molecular interfaces of the binding epitopes for both type I and type II receptors, sequestering the covalently linked homodimeric ligand in an inactive complex [13]. In the crystal structure of GDF5 [27], the conserved asparagine (N445) is located near the amino terminal end of the large helix (α3), its amide sidechain projecting tangential to, and hydrogen bonding with, the backbone of the long finger 2 at a main chain carbonyl (E491) across the dimer interface. Superposition of the GDF5 crystal structure on BMP7 in the NOG-BMP7 complex provides a model of the NOG-GDF5 complex for interpretation of the role of the conserved asparagine and the consequences of the mutations. In the model, just as in the NOG-BMP7 crystal structure, the amide nitrogen of the asparagine sidechain is hydrogen bonded to two main chain carbonyl groups, one across the dimer interface (GDF5 E491) and one along the extended N-terminal 14 clip of the antagonist (NOG A36). In addition to this triangulated interaction, the carbonyl oxygen of the N445 sidechain also forms hydrogen bonds with the main chain via the amide nitrogen of NOG A36. Furthermore, in a superposition model of a GDF5-type I receptor complex, the asparagine sidechain appears to interact with E81 and G82 across the ligand-receptor interface. Thus, loss of the amide group by substitution with a lysine or threonine side chain in the N445K,T mutants would on the one hand disrupt two stabilizing interactions within the NOG-GDF5 complex, and, on the other hand, alter the ligand-receptor interface resulting in a broader specificity. With respect to the differential effects of threonine substitution on interaction with NOG, superposition of GDF5 and BMP7 in the crystal structure of the BMP7-NOG complex reveals a major structural difference between the two ligands at the interface with the key residue of the N-terminal extension of NOG, P35, also a site of several human mutations linked to SYM1 [13],[28]. Due largely to major conformational differences in the backbone and side chains of the short finger 1 of GDF5 relative to the BMPs, the hydrophobic pocket that accommodates the proline side chain is predicted to be only half formed, which would disrupt the stabilizing interaction to a similar extent as mutation of P35. Thus, interaction between wildtype GDF5 and NOG is anticipated to be weaker than that between BMPs and NOG, as evidenced by our titration assays which required super-stoichiometric ratios (*cf.* Figure 5B), perhaps so much so that disruption of the adjacent interaction between the conserved asparagine and the backbone of the N-terminal extension (A36) is sufficient to abolish formation of the GDF5-NOG complex. BMP9 and BMP10, which are not responsive to inhibition by NOG, do not bear the homologous asparagine residue. However introduction of the asparagine in BMP9 was not sufficient to confer sensitivity to NOG. Successive introduction of two additional GDF5-like substitutions, removal of a BMP9-specific insert (YH415Q) and K348L, imparted near total sensitivity. In conclusion, while substitution of the single asparagine is sufficient to impart NOG sensitivity in GDF5, other BMPs require further structural alteration in order to impart sensitivity, or resistance. Because ligand-receptor and ligand-antagonist complexes are sufficiently structurally conserved within the BMP/GDF family, GDF5 function was successfully transferred to BMP9 by rounds of rational design.

Currently, rhBMP2, rhBMP7, and rhGDF5 are used in clinical applications for bone regeneration or are under investigation in clinical trials. Although BMPs are very potent in cell culture systems or small animal models (nanograms), significantly higher concentrations are needed for measurable effects in humans (milligrams). It was shown that BMP antagonists are regulated during fracture healing [29], indicating that endogenous regulation of the BMP signaling cascade may neutralize exogenously applied rhBMPs, necessitating higher doses than required in vitro. In keeping with this hypothesis, the in vivo and in vitro studies presented here demonstrated induction of Nog after treatment with rhBMPs, an apparent physiological response to regulate and constrict BMP action. The upregulation of inhibitors in response to exogenous growth factors may therefore also be applied for BMP treatment, which can only be partially compensated for by high dosage due to feedback control mechanisms [9], [30]–[31]. Thus, variant BMPs that are insensitive to antagonist may induce bone formation more effectively, providing a source for effective, low-dose therapeutics for clinical applications.

## Materials and Methods

### Patients and molecular analysis

All clinical investigations have been conducted according to Declaration of Helsinki principles. The study was approved by the local institutional review board. Patients were investigated and radiographed by a clinical geneticist who diagnosed typical SYNS1. Informed consent was obtained for genetic analyses from all patients or the legal guardians. Mutation screening in *GDF5* was carried out as previously described on purified DNA obtained from blood sample [32].

### Alignment and 3D structure modeling

Protein sequence alignments comprising the highly conserved cysteine knot domains of the human TGFβ superfamily were aligned using CLUSTAL X (http://bio.ifomfirc.it/docs/clustal/clustalx.html) [33] and colored using CHROMA (http://www.lg.ndirect.co.uk/chroma/) [34]. GDF5-NOG complex modeling was previously published [28]. Images of the molecular structure were produced using the UCSF Chimera package (http://www.cgl.ucsf.edu/chimera/) [35].

### Cloning and production of retroviruses

The coding sequences of mouse (m) *mBmp9*, *mBmp10* were amplified by PCR on mouse embryo E14.5 cDNA and cloned into pSLAX-13 using the following primer pairs: *mBmp9*_BsmBI_f ACGTCTCCCATGTCCCCTGGGGCCTTCCG and *mBmp9*_BamHI_r TGGATCCTACCTACACCCACACTCA, *mBmp10*_BsmBI_f GATACGTCTCCCATGGGGTCTCTGGTTCTGCC and *mBmp10*_XmaI_r GATCCCCGGGCTATCTACAGCCACACTCAGAC. In vitro mutagenesis for chicken (ch) *chGdf5* and *mBmp9* was performed with Quickchange Kit (Stratagene) according to manufacturer's recommendations. Cloning into retroviral vector, and production of viral supernatant in DF1 cells and concentration of viral particles was performed as described [36]. RCAS(BP)B-*Nog* was a kind gift of Andrea Vortkamp.

### Recombinant proteins

RhGDF5 (Biopharm GmbH) was dissolved in 10 mM HCl, rhNOG was a kind gift from A. Economides (Regeneron Pharmaceuticals Inc.) and dissolved in 0.1% BSA/PBS, rhBMP2 was a kind gift from W. Sebald and dissolved in 4 mM HCl, 0.1% BSA, rhBMP9 (R&D Systems) was dissolved in 4 mM HCl, 0.1% BSA. RhN445T GDF5 was prepared as previously described with minor modifications [16]. Inclusion bodies were dissolved in 8 M Urea, 20 mM Tris HCl, 5 mM EDTA, 50 mM NaCl, and 66 mM DTT, pH 8.3. Purification was carried out on a cation exchange column SP Sepharose FF (XK26/40, GE Healthcare) with a gradient from 100% eluent A (6 M Urea, 20 mM Tris HCl, 1 mM EDTA, 50 mM NaCl, 10 mM DTT, pH 8.3) to 100% eluent B (6 M Urea, 20 mM Tris HCl, 1 mM EDTA, 400 mM NaCl, 10 mM DTT, pH 8.3), flow rate 3.5 ml/min. Eluents were concentrated by ultrafiltration (Amicon, Omega 5) and adjusted to 0.5 M NaCl. This solution was dissolved 1∶10 in refolding buffer (150 mM NaGlycine, 500 mM NaCl, 20 mM 33 mM 3-(3-cholamidopropyl) dimethylammonio-1-propanesulfonate, 3 mM oxidized glutathione (GSSG), 1 mM EDTA, pH 9.8) under gentle agitation so that a final protein concentration of 1 mg/ml was reached. The solution was then isoelectric precipitated with 1.8-fold 20 mM NaH_2_PO_4_ at 4 °C for 1 hour, pH 7.4. After centrifugation the pellet was dissolved in 0.1% trifluoroacetic acid. The dimeric GDF5 N445T was separated from monomeric protein by RP-HPLC (reversed phase column Source 15 RPC, fine line pilot 19 35/20 cm, GE Healthcare) with a gradient from 100% eluent A (0.1% trifluoroacetic acid (TFA)) to 100% eluent B (0.1% TFA, 90% CH3N), flow rate 14 ml/min.

### Micromass cultures

Micromass cultures were performed as previously described [16]. For each condition, four replicates were performed in parallel and every experiment was done three times. Stimulation of micromass cultures with rhGDF5, rhN445T GDF5, rhBMP2, and rhBMP9 was performed at day one for 12 or 72 h.

### Quantitative real-time PCR

Micromass cultures were lysed in Trifast (peqlab) after 12 h of stimulations with recombinant human proteins and total RNA was isolated according to the manufacturer's recommendations. cDNA synthesis was performed with Taqman Kit (Applied Biosystems) according to manufacturer's guidelines. Gene expression was assessed by amplification of Nog and β-Actin as endogenous control on a Taqman 7500 (ABI) using SYBR green. The following primer pairs were used: chNog-526f TCTGTCCCAGAAGGCATGGT, chNog-590r CGCCACCTCAGGATCGTTAA, chActin-410f CAACAGAGAGAAGATGACACAGATCA, chActin-484r ACAGCCTGGATGGCTACATACA.

### Alkaline phosphatase (ALP) activity on different mouse cell lines after BMP stimulation

The myoblastic mouse cell line C2C12 (ATCC) and C2C12 stably overexpressing Bmpr1b (C2C12-Bmpr1b; [37]) were seeded in 96-well plates at a density of 1.5×10^4^/well in growth medium (high-glucose DMEM, 10% FCS, and 2 mM L-Gln in 10% CO_2_). 24 h later they 20 were stimulated in starved medium (2% FCS) for 72 h with recombinant proteins. The measurement of ALP activity was performed by pNPP as previously described [16].

### Biacore experiments

The BIA2000 system (Biacore) was used to analyze the binding affinity of rhGDF5 and rhN445T GDF5 to immobilized receptor ectodomains of BMPR1A and BMPR1B as described previously [16].

### Luciferase reportergene assay

The luciferase reportergene assay was performed with minor changes as previously described [38]. C2C12 cells were grown in DMEM high glucose with 10% FCS. SBE-pGL3 [39] and pRL-Tk (Promega) transfected C2C12 cells were seeded at a density of 1*10^5^ cells/96-well and stimulated one day later for 15 hours with recombinant proteins in DMEM high glucose with 2% FCS. Luciferase activity was determined using the Dual-Glo Luciferase Reporter Assay System (Promega).

### In vivo manipulations of chicken limb buds

Injections of RCAS(BP)A viruses into HH10 chick embryo hind limb fields was performed as previously described [36],[40]. Afterwards embryos were harvested at day 7.5 (HH31–33) and fixed in 100% ethanol. Skeletal preparation and staining was performed with Alcian blue and Alizarin red [40]. Every construct was injected at least in 60 embryos. The effect of rhGDF5, rhN445T GDF5, rhBMP2, and rhBMP9 in vivo on *Nog* expression was determined by implantation of heparin-acrylic beads (Sigma-Aldrich) soaked with recombinant human proteins in chicken limb buds. The beads were implanted between stages HH25–27, in case of rhBMP9 at stage HH29/30 as described [30]. After 20 to 22 h the embryos were harvested and fixed in PFA. Every protein was implanted at least in 10 embryos.

### In situ hybridization

Fixation of chicken embryos was performed with 4% PFA. Whole mount in situ hybridization was performed as previously described [41]. DIG-labeled probe of *chNog* [42] was detected with BMPurple (Roche).
